# Multifrequency Vector Measurement System for Reliable Vehicle Magnetic Profile Assessment

**DOI:** 10.3390/s20174933

**Published:** 2020-08-31

**Authors:** Zbigniew Marszalek, Krzysztof Duda

**Affiliations:** Department of Measurement and Electronics, AGH University of Science and Technology, 30 Mickiewicz Avenue, 30-059 Krakow, Poland; kduda@agh.edu.pl

**Keywords:** auto-balancing bridge method, FIR filter, FPGA, impedance, inductive-loop sensor, multifrequency, vehicle magnetic profile, vector voltmeter, signal processing

## Abstract

This paper describes the design and the performance of simultaneous, multifrequency impedance measurement system for four inductive-loop (IL) sensors which have been developed for vehicle parameters measurement based on vehicle magnetic profile (VMP) analysis. Simultaneous impedance measurement on several excitation frequencies increases the VMP measurement reliability because typical electromagnetic interferences (EMI) are narrowband, and should not simultaneously affect, in the same way, all measurement bands that are spread in the frequency, i.e., it is expected that at least one measurement band is disturbance-free. The system consists of two standard and two slim IL sensors, specially designed and installed, the analogue front-end, and an industrial computer with digital-to-analogue and analogue-to-digital converters accessed via field-programmable gate array (FPGA). The impedance of the IL sensors is obtained by vector measurement of voltages from auto-balancing bridge (ABB) front-end. Complex voltages are demodulated from excitation frequencies with FIR filters designed with the flat-top windows. The system is capable of delivering VMPs in real-time mode, and also storing voltages for off-line postprocessing and analysis. Field distributions and sensitivities of slim and standard IL sensors are also discussed. Field test confirmed assumed increased reliability of VMP measurement for proposed simultaneous multifrequency operational mode.

## 1. Introduction

Over the last several decades, inductive-loop (IL) sensors have become increasingly important as reliable vehicle detectors, for proper operation of traffic control systems, as well as for increase of the responsiveness of intelligent transportation systems (ITS) [1]. However, a multifrequency impedance measurement is a new uprising research filed in the IL sensor technology.

Currently, there are many technologies available for measurement of vehicle parameters in traffic, such as vehicle speed, weight, length, its number of axles, the distance between the axles, vehicle class, and so on [2]. The most common sensors used for traffic measurement are inductive, hydraulic, magnetic, pneumatic, reluctance, resistive, optical fiber, capacitive, laser, piezoelectric, quartz, tensometric, acoustic, ultrasonic, microwave and infrared, light curtains, and cameras with image processing algorithms [3,4,5].

When it is necessary to detect the vehicle axles, and to measure their arrangement in the vehicle body, in principle, above group limits down to sensors providing signals associated with vehicle wheels. It can be accomplished by a proper set of load sensors, i.e., piezoelectric, quartz, tensometric, fiber [6], and at least one standard, i.e., 1 m by 2 m, IL sensor for grouping axles of a given vehicle [7]. Systems equipped with load sensors provide accurate measurements of the axles number and the distance between them. Load sensors usually dedicated to weigh-in-motion (WIM) applications [8], have a nominal two years working life and thus are not a long-life neither low-cost solution. Load sensors differ in price, installation method, and operating principle, but they all have the same drawback as their active element is exposed to the force exerted by the tires of the passing vehicle. This main drawback not only limits the load sensor lifespan, but can also lead to the misclassification of vehicles with lifted axles [9]. In addition to load sensors, WIM systems must also have at least one IL sensor for vehicle detection.

The measurement system consists of an IL sensor, connecting cables, controller cabinet, and detector electronics. The IL sensor is made from a several loops of isolated wire wound permanently mounted in the pavement of a road. The IL sensor is powered by the detector electronics at frequencies typically ranging from 10 to 200 kHz [10]. The IL sensor is and inductive element of a tuned electrical circuit. A vehicle passing over the IL sensor affects its inductance. This change of inductance activates the detector electronics, which further initialize the controller unit. Direct measurement of the inductance change is not possible because signal conditioning systems are based on oscillating circuit [10]. The inductance change causes phase and frequency shifts in the output signal [11].

Advanced IL system can provide an inductance waveform when a vehicle passes over the sensor. This waveform, known as vehicle signature, or vehicle magnetic profile (VMP), can be used in vehicle re-identification, and matching vehicles between an upstream and downstream location [12,13].

The systems presented in [10,11,12,13] contain structurally different IL sensors, but they all share common characteristics. The IL sensor works together with a circuit, where the inductance change is measured indirectly by frequency change.

The frequency change of oscillating circuit, i.e., the magnetic profile, contains only the information associated with the inductance of IL sensor. Due to the operational principle of a system based on any resonant circuit, the information associated with the real part of IL impedance, i.e., the resistance, is lost [14]. The loss of resistance waveform leads to limitations in the development of signal processing algorithms based on electrical knowledge.

A different approach to the vehicle magnetic profile assessment is used in the AC-bridge-based system [7], where the impedance components (real and imaginary) in the output are irreversibly mixed together and the mixing depends on the set of synchronous demodulator phase angle.

There are axles detection algorithms based on both resistance and inductance waveform of IL sensor impedance components [9]. However, these algorithms cannot work on the inductance waveform delivered by the system based on oscillating circuit technology. For this reason, a method for obtaining both resistance and inductance waveform from one IL sensor was carried out [9,15].

A vehicle generates strong electromagnetic interference (EMI) in the IL sensors field, with the spectrum overlapping the operational band of impedance measurement unit (IMU), which essentially deteriorate the performance of the IMU with the impedance components separation [16]. In the proposed measurement system, the IL sensor impedance is acquired on several frequencies simultaneously [17,18,19,20], which reduces the disturbing influence of a vehicle engine EMI. The impedance waveforms with detected disturbances are then rejected before further processing.

We propose a new approach to impedance waveform measurement. The novelty of our solution is threefold: (1) Applied IL sensors are arranged in original quad configuration consisting of two slim loops and two standard loops. (2) The designed impedance measurement system works simultaneously in three separate frequency bands. (3) A new application of auto-balancing bridge (ABB) method [21] is presented.

Fully functional measurement system was built and validated via extensive field tests. The IL sensors were mounted in the real road near a parking lot. Early signal processing was realized using National Instrument PXI industrial system. Obtained results confirmed overall performance of the proposed measurement system.

The paper is organized as follows. Section 2 presents the proposed IL quad sensors arrangement, sensor model, and sensitivity description of the slim and standard loop. Section 3 describes the multifrequency IMU followed by system hardware implementation and signal processing stage. Section 4 describes the experimental set-up and the experimental results. Section 5 concludes the paper.

## 2. Applied IL Quad Sensors

### 2.1. Arrangement and Dimensions of IL Sensors

Vehicles, as metal objects, passing through the alternating magnetic field of IL cause changes to the IL impedance both in the real and imaginary part. These changes, on the level of a few percent of IL nominal impedance value, provide useful information for algorithms measuring vehicle parameters in traffic.

Changes in the monitored values of impedance represented as a function of time during the vehicle passing over the IL are called the vehicle magnetic signature [1] or VMP.

**Figure 1:**
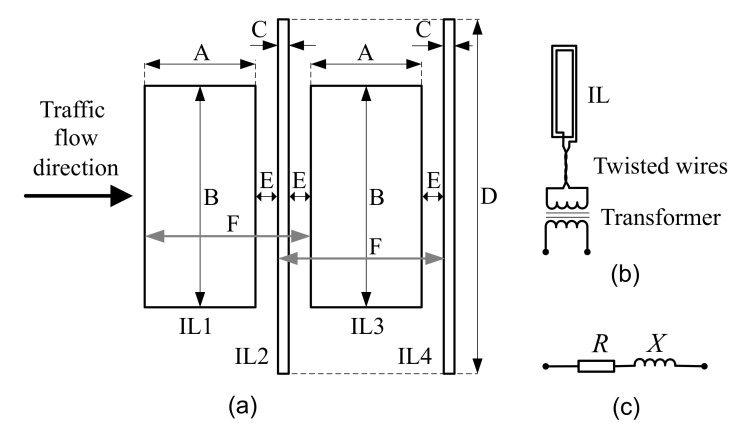
Proposed inductive-loop (IL): (**a**) sensors arrangement on lane. Dimensions are specified in Table 1. (**b**) Hardware components and (**c**) serial model.

The proposed quad IL arrangement, presented in Figure 1, consists of two standard loops—IL1 and IL3—and two slim loops—IL2 and IL4. Standard loops work as typical dual-loop vehicle detectors [5]. Slim loops are dedicated for axle detection [9] and distance measurement between them.

Dimensions of loops are defined in Figure 1 and given in Table 1. The distance between standard loops, IL1 and IL3, is 0.5 m, and the distance between slim loops, IL2 and IL4, is 1.4 m. This arrangement of compact loops takes 2.8 m in a drive direction. Due to the purpose of the slim loops, they are longer than standard loops (compare dimension B and D). The distances, F, between the front edges of the standard and slim loops are the same.

Standard loops, IL1 and IL3, are made with 4 turns of wire, and slim loops, IL2 and IL4, are made with 8 turns of the same wire with a cross section of 2.5 mm^2^.

**Figure 2:**
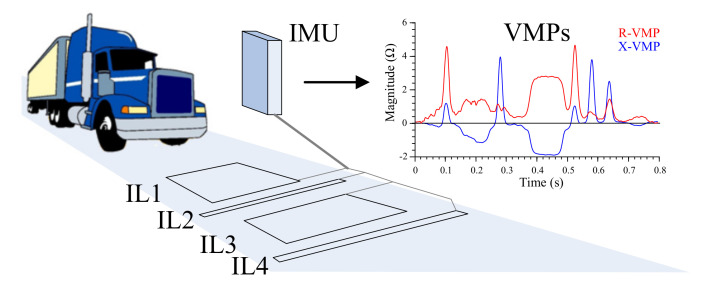
Inductive loop (IL) sensors arrangement on lane, impedance measurement unit (IMU), and vehicle magnetic profiles (VMPs) example from IL2 for a single frequency.

Figure 2 presents quad IL mounted in the road. They have the following advantages; the ability to measure vehicle speed [5], length, the direction of movement, and distance between axles as well as the number of axles even if they are lifted. Suspension height as well as front and rear overhang of the vehicle are also possible to estimate.

### 2.2. Galvanic Isolation and IL Sensor Model

The distance between the arrangement of IL sensors and cabinet for IMU may vary from tens to a hundred meters. In measurement practice, a single IL sensor consists of a loop installed on the lane, long twisted connection wires, and a transformer for galvanic separation, see Figure 1b. Due to the long underground installed connection wires, galvanic separation is absolutely necessary to protect the IMU. Although the sensor consists of several elements arranged over a large area, a serial equivalent circuit model of impedance is used to describe the sensor, as shown in Figure 1c.

The transformer for galvanic separation is made with a ferrite core and its transmission ratio is approximately 1:7. Therefore, the nominal impedance of IL with the transformer is higher than the loop impedance. Examples of measured impedances, using E4980A instrument [22], are shown in Table 2.

### 2.3. The Principle of IL Sensor Operation

The flow of AC current through the IL causes a magnetic field around the winding. According to Maxwell’s equations, eddy currents in a conductive metal object present in the field change the global magnetic field distribution. There are dynamic interactions of eddy currents field and inductive loop magnetic field. If the IL impedance is monitored, the effect of these interactions can be seen as impedance changes.

### 2.4. Field Distribution and Sensitivity of IL

This section describes the sensitivity of the IL in relation to the magnetic field distribution generated by the loop. A sensitivity indicates how much the sensor’s output changes with the change of the input measured quantity. Therefore, to characterize the sensitivity of the IL, which is related to its magnetic field distribution, it is necessary to visualize the distribution of its field in the active space that is penetrated by vehicles. The distribution of the field can be calculated using the Biot–Savart law and presented on the plane in selected cross-sections [1].

The wheel rim is the closest part of the car that is extended towards the IL sensor. The distance between the rim and the loop wires depends on the height of the tire (e.g., 8 cm) and the mounting depth of IL in the road (e.g., 2 cm). Therefore, as an example, the magnetic field induction distribution in the air, over the IL, in a horizontal section in a plane parallel to the sensor, located at a distance of 10 cm was computed. For comparison, the constant dimensions of spatial cross-sections for slim and standard IL sensors, and the same current flow of 1 A-turn were adopted.

**Figure 3:**
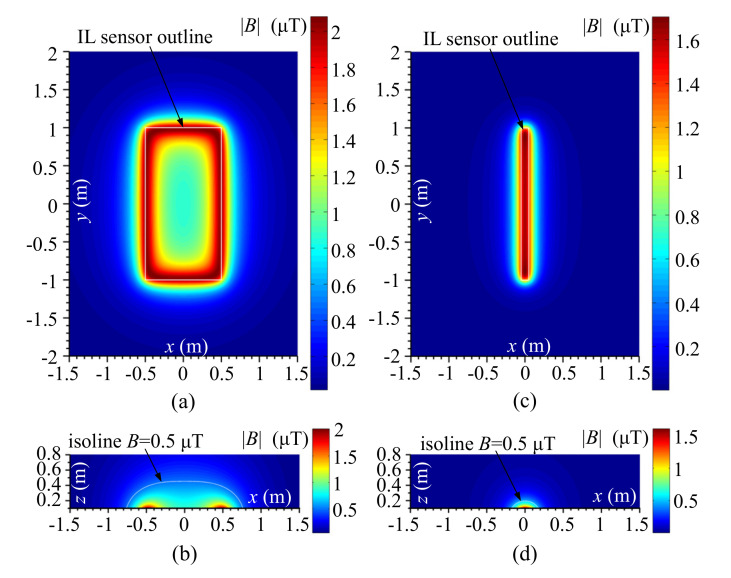
Magnetic field module distribution, |B| (µT), of the standard-loop: (**a**) 10 cm above the plane of the IL wires and (**b**) in vertical cross section. The slim-loop (**c**) 10 cm above the plane of the IL wires and (**d**) in vertical cross section.

The field distribution, in a horizontal plane, 10 cm above the standard loop, is shown in Figure 3a,b, which also shows the field distribution in the vertical cross plane. In Figure 3b, the isoline *B* = 0.5 µT is marked, with the magnetic induction value 4 times smaller than the maximum magnetic induction in the considered area.

Field distributions for the slim loop were calculated analogically and are shown in Figure 3c,d. By comparing and analyzing IL sensors field distributions, one can come to the conclusion that standard loops generate a spread field, which simultaneously includes many vehicle chassis components. The induction values, not less than 0.5 µT, occur where there are highly located vehicle chassis components, e.g., trucks. This explains why standard loops are suitable for detecting entire vehicle bodies, even those with high suspension.

The different situation is in the case of the slim IL sensors because their field is spatially less spread. Values higher than 0.5 µT, see Figure 3d, are focused close to the loop. Thus, the slim-loop sees less than the standard-loop. It is stated that the slim-loops also have a lower range of their magnetic field. Thanks to this feature, slim-loops are dedicated to wheel rims detection, which allows getting information about the number and arrangement of axles in the vehicle body.

The field distribution reveals the sensors features, which have an effect on the resultant sensitivity of the loops considered. The standard-loop is sensitive to objects in a much larger space above the IL than the slim-loop, which exhibits scanning properties of details protruding from large objects such as metal parts of vehicle wheels, rocker arms, bumpers, etc.

The presented field distributions also indicate that the measurement system will not work properly only with slim-loop. It must be equipped with standard-loops providing strong information about the vehicle body when the slim-loop is between the axles and its field does not reach to high suspensions elements. The slim IL sensor used for vehicle axle detection requires more sensitive, and more sophisticated IMU.

## 3. Multifrequency Impedance Measurement System

This section describes the multifrequency, 4-channel impedance measurement system able to estimate VMPs simultaneously in three different measurement bands for each channel. The system features auto-balancing operation, i.e., the need of time-consuming balancing during the absence of the vehicle in IL field is eliminated. The VMP amplitude resolution was increased by oversampling and averaging [23]. A greater number of samples also improves noise properties of the frequency estimator. The variance of amplitude estimation of a sinusoid disturbed by additive white Gaussian noise is inverse proportional to the number of samples, and the variance of frequency estimation is inverse proportional to the third power of the number of samples [24,25]. The power of quantization noise do not depend on sampling frequency and noise spreads, in the frequency domain, form 0 to the half of the sampling frequency. Thus, by increasing the sampling frequency the noise level is lowered, and the signal to noise ratio in the sub-band of interest is increased [26]. The highest possible sampling frequency enabled in the PXI system, that is, 400 kHz, was used. During amplitude and phase demodulation signals are averaged by FIR filter with the impulse response lasting 0.1 s.

### 3.1. Analogue Section

**Figure 4:**
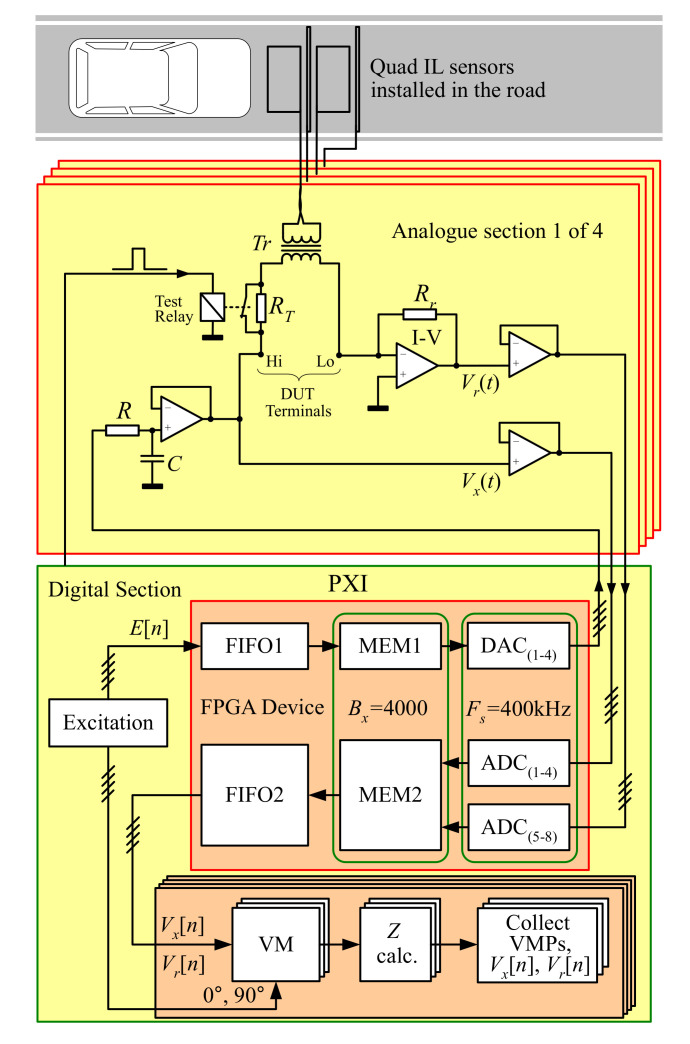
The hardware design of the field-programmable gate array (FPGA)-based four-channel system for multifrequency impedance measurement of IL sensors, where VM is a vector measurement.

The measurement system, used for the complex impedance of the device under test (DUT) acquisition, consists of a sinusoidal excitation source, a vector voltmeter, and a vector ammeter. The auto-balancing bridge (ABB) method is commonly used in modern single-frequency impedance measurement instruments [21]. The ABB circuits used for low-frequency impedance measurement (below 100 kHz) typically exploit a basic current-to-voltage (I–V) converter and an operational amplifier with a negative feedback, as depicted in Figure 4. The DUT consists of IL sensor, connecting wires, and transformer.

The digital-to-analogue converter (DAC_1_) is used to digitally generate the voltage excitation signal $V_{x}\left(t\right)$, that is next filtered by an analog low-pass filter, and then excites the current flow through the DUT and the I–V converter. The same current, as the one flowing through the DUT, is derived by the operational amplifier, of the I–V converter, in the negative feedback loop. The potential at the Lo terminal of the DUT is automatically balanced to zero because the feedback current flowing through the $R_{r}$ is equal to the input current [21,27].

The vector voltages, $V_{x}\left[n\right]$ and $V_{r}\left[n\right]$, are calculated in Section 3.2. As the $R_{r}$ value is known, in static condition, to calculate complex impedance Z[n] of the DUT, we use:

(1) $$Z\left[n\right]=R\left[n\right]+jX\left[n\right]=R_{r}\frac{V_{x}\left[n\right]}{V_{r}\left[n\right]}$$

where *n* is the number of the sample.

It should be noted that $V_{x}\left[n\right]$ and $V_{r}\left[n\right]$ are discrete-time versions of continuous-time counterparts $V_{x}\left(t\right)$ and $V_{r}\left(t\right)$. Different digital signal processing algorithms can be used in quadrature demodulation process for obtaining complex-valued voltages $V_{x}\left[n\right]$ and $V_{r}\left[n\right]$ used in (1).

For example, in [27] a simple vector voltmeter algorithm for $V_{x}\left[n\right]$ and $V_{r}\left[n\right]$ calculation for the individual frequencies and in static conditions was used.

In this work, signals $V_{x}\left[n\right]$ and $V_{r}\left[n\right]$ are demodulated simultaneously on all excitation frequencies by complex-valued FIR filters, as explained later in the paper.

The correct system operation on all channels, and on all measurement bands simultaneously is validated by a non-inductive test resistor, $R_{T}=1\;\Omega$ installed in series with the transformer Tr. Test resistor is normally bypassed via test relay. The validation of the system operation is based on the short switching the relay and observation of all outputs. Changes visible in the real part of measured impedance, increment equal to one ohm, and also no changes in the imaginary part, confirm the correct overall operation of the system.

### 3.2. Digital Section

The PXI system with the NI-RIO field-programmable gate array (FPGA) circuit is used for implementation of Section 3.2. The FPGA module contains also digital-to-analog converters ${\mathrm{DAC}}_{\left(1-4\right)}$, analogue-to-digital converters ${\mathrm{ADC}}_{\left(1-8\right)}$, and data memory for signals MEM1 and MEM2 with a fixed size of $B_{x}$ = 4000 samples per signal block. The data can be written to memory MEM1 and MEM2, and read from this memory, by FIFO queues via DMA channels. The DACs and ADCs work synchronously with the sampling frequency of $F_{s}$ = 400 kHz. FPGA transmits 100 blocks of signals per second to the host. The system has been implemented in LabVIEW.

As shown in Figure 4, FPGA supports digital-to-analog and analog-to-digital conversion, whereas the main system CPU is used for excitation generation and impedance parameters calculation and acquisition. Considering the available hardware resources, it is expected that the demodulation can fully be implemented in FPGA, what will be the subject of further work.

The basic configuration of the system hardware is described in Table 3.

### 3.3. Excitation

The excitation voltage E[n] is a *K*-sine discrete-time signal that can be defined in general form by

(2) $$E\left[n\right]=\sum_{k=1}^{K}A_{k}\sin\left(\omega_{k}n+\varphi_{k}\right)$$

where $\omega_{k}=2\pi F_{k}/F_{s}$ is a normalized pulsation in radians of a discrete-time signal, $F_{s}$ is the sampling frequency in hertz, $A_{k}$ is the amplitude, $F_{k}$ is the frequency in hertz, $\varphi_{k}$ is the phase angle in radians, the lower subscript *k* refers to the *k*t frequency component of the excitation signal, and *n* stands for the sample number.

Amplitudes $A_{k}$ may be adjusted to provide expected DUT current for given frequency [17]. The phase angles $\varphi_{k}$ have been properly selected to minimize the crest factor [18].

To avoid discontinuities in the excitation signal (2), excitation packets include an integer number of sinusoidal cycles of each frequency and are converted to analog signals without any transitions effects, i.e., obtained continues-time signals are smooth. As long as the block of excitation signal $B_{x}$ has the length of 4000 samples and the sampling frequency $F_{s}$ is 400 kHz, we obtain the minimum frequency spacing $f_{d}=F_{s}/B_{x}$ ($f_{d}=$ 100 Hz) between working frequencies.

### 3.4. Signal Demodulation

Discrete-time measurement signals $V_{x}\left[n\right]$ and $V_{r}\left[n\right]$ are bandpass signals with amplitude and phase modulation in the form

(3) $$V_{x}\left[n\right]=\sum_{k=1}^{k}A_{k}^{x}\left[n\right]\sin\left(\omega_{k}n+\varphi_{k}^{x}\left[n\right]\right),$$

where the upper subscript *x* refers to the signal $V_{x}\left[n\right]$. With the symbol *x* replaced by *r* in (3) we get the model valid for $V_{r}\left[n\right]$. Signals $V_{x}\left[n\right]$ and $V_{r}\left[n\right]$ are demodulated simultaneously on all excitation pulsations $\omega_{k}$ by means of the flat-top bandpass Hilbert transformers (FTBPHT) implemented as FIR filters [28]. FTBPHT was chosen due to exceptional accuracy of amplitude and phase demodulation, and high rejection of unwanted spectral components, e.g., other carrier frequencies. Complex-valued impulse response $h_{k}\left[n\right]$ of FTBPHT FIR filter is obtained by modulating the flat-top window $w_{M}\left[n\right]$ to the desired excitation pulsation $\omega_{k}$

(4) $$h_{k}\left[n\right]=w_{M}\left[n\right]e^{j\omega_{k}n},$$

where the flat-top window is the following cosine window [29],

(5) $$w_{M}\left[n\right]=\left\{\begin{array}{c} \sum_{m=0}^{M}c_{M}\left[m\right]\cos\left(m\frac{\pi }{N}n\right),\;n=-\frac{N-1}{2},\ldots ,\frac{N-1}{2}\\ 0,\;\mathrm{otherwise} \end{array}\right.$$

where *M* is the window order, $c_{M}\left[m\right]$ are coefficients of an *M*-order window, and *N* is odd window length. We used the 3rd order flat-top window with coefficients $c_{3}\left[0\right]$ = 1, $c_{3}\left[1\right]$ = 1.9918, $c_{3}\left[2\right]$ = 1.7038, $c_{3}\left[3\right]$ = 0.71199, and length *N* = 40,001 samples.

**Figure 5:**
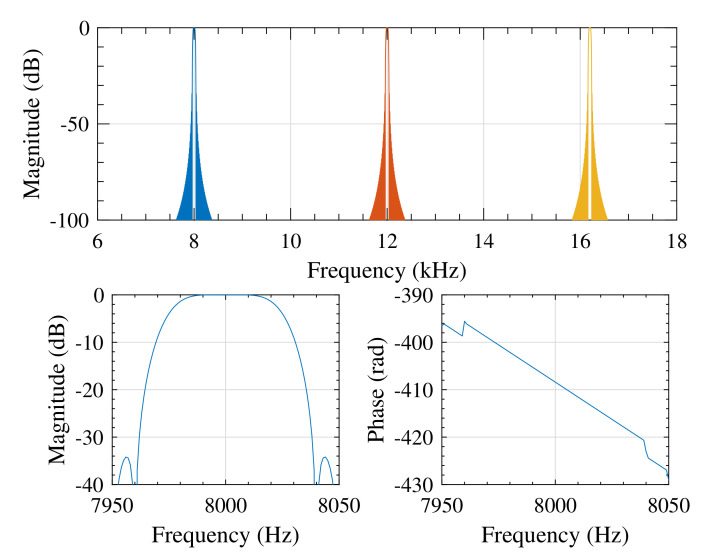
Frequency magnitude responses of the flat-top bandpass Hilbert transformers (FTBPHTs) configured for three working frequencies $f_{1}$ = 8 kHz, $f_{2}$ = 12 kHz, and $f_{3}$ = 16.2 kHz (top), and zoomed magnitude and phase response for the FTBPHT working with $f_{1}$ = 8 kHz (bottom). The length of each FTBPHT filter is 40,001 samples. The shapes of magnitude and phase responses of the remaining two filters in passbands are approximately the same.

Figure 5 presents frequency responses of three FTBPHTs configured for excitation signal containing three sinusoidal components with frequencies $f_{1}$ = 8 kHz, $f_{2}$ = 12 kHz, and $f_{3}$ = 16.2 kHz.

The decaying of the side lobes of the filters is very fast thus for a specific excitation component high robustness against other frequency components, including remaining excitations, is ensured. The passband of each FTBPHT is flat what gives some margin for excitation frequency fluctuation. For the signal $V_{x}\left[n\right]$ (3), the output of the *k*th FTBPHT is

(6) $$v_{k}^{x}\left[n\right]=A_{k}^{x}\left[n\right]e^{j\varphi_{k}^{x}\left[n\right]},$$

and similarly for the signal $V_{r}\left[n\right]$.

### 3.5. Implementation of Vector Measurement

The output (6) of the *k*th FTBPHT is the convolution sum of measurement signals $V_{x}\left[n\right]$, $V_{r}\left[n\right]$ (3) and the complex-valued filter impulse response $h_{k}\left[n\right]$ (4)

(7) $$v_{k}^{x}\left[n\right]=\sum_{m=0}^{N-1}h_{k}\left[m\right]V_{x}\left[n-m\right]$$

and similarly for $v_{k}^{r}\left[n\right]$, with indexes *x* replaced with *r* in (7).

Measurement signals $V_{x}\left[n\right]$ and $V_{r}\left[n\right]$ are heavily oversampled narrowband signals, and the filter outputs $v_{k}^{x}\left[n\right]$ and $v_{k}^{r}\left[n\right]$ may be computed every *L* samples instead of every single sample. By substituting n=lL, *l* = 0, 1, 2, … in (7) the filter $h_{k}\left[n\right]$ is shifted by *L* samples along the filtered signal which *L* times reduces computational effort. The impedance value over time for *k*th excitation component is computed by

(8) $$Z_{k}\left[n\right]=R_{k}\left[n\right]+jX_{k}\left[n\right]=R_{r}\frac{v_{k}^{x}\left[n\right]}{v_{k}^{r}\left[n\right]}.$$

## 4. Results

The proposed four-channel system for simultaneous, multifrequency impedance measurement of IL sensors was built using FPGA and tested. In every single channel, three unique excitation frequencies have been applied. Taking into account the physical properties of the measurement system and its environment, and also the previous test and simulation results [16], the frequency range from 6 to 17.2 kHz with spacing of 4 kHz was adopted. This range of frequencies ensures acceptable measurement sensitivity and disturbance immunity. The list of excitation frequencies is presented in Table 4.

The sampling frequency of the ADC and DAC converters was set to $F_{s}$ = 400 kHz. For signal demodulation the $h_{k}\left[n\right]$ filter length *N* = 40,001 coefficients, and the downsampling parameter of *L* = 400 samples was set. In this set-up configuration, the system outputs 1000 samples of impedance per second for a single excitation frequency, which in overall is 1000 × 4 × 3 samples for 4 IL sensors each working on 3 excitation frequencies.

Computed VMPs ($R_{k}\left[n\right]$, $X_{k}\left[n\right]$) are adjusted to begin with the value equal zero for presenting in common plots.

**Figure 6:**
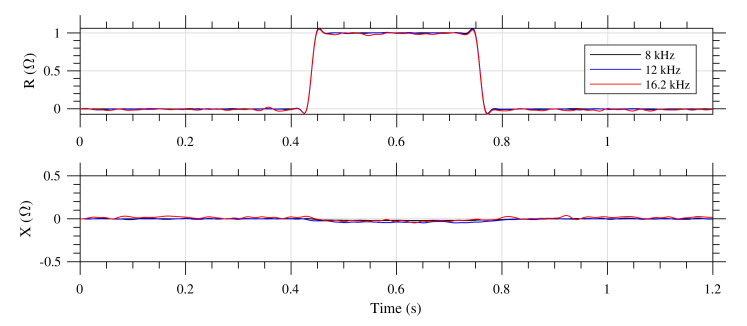
The test-relay system operation validation results for the first standard IL1 sensor channel. On the top, there is the R component, bottom X, and the legend describes the frequencies, see Table 4.

Figure 6 shows the system operation validation results for the first standard IL1 sensor channel during the short switching the relay (see Figure 4). Changes visible in the real part of measured impedance signals (R), increment equal to 1 Ω, and also negligible changes in the imaginary part (X), confirm the system correct operation.

**Figure 7:**
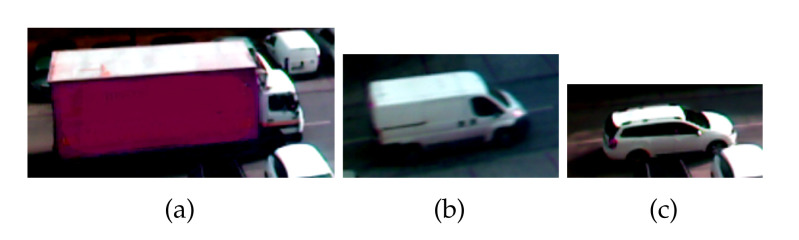
Vehicles for which VMPs were registered and analyzed: (**a**) a truck, (**b**) a delivery car, and (**c**) a passenger car.

**Figure 8:**
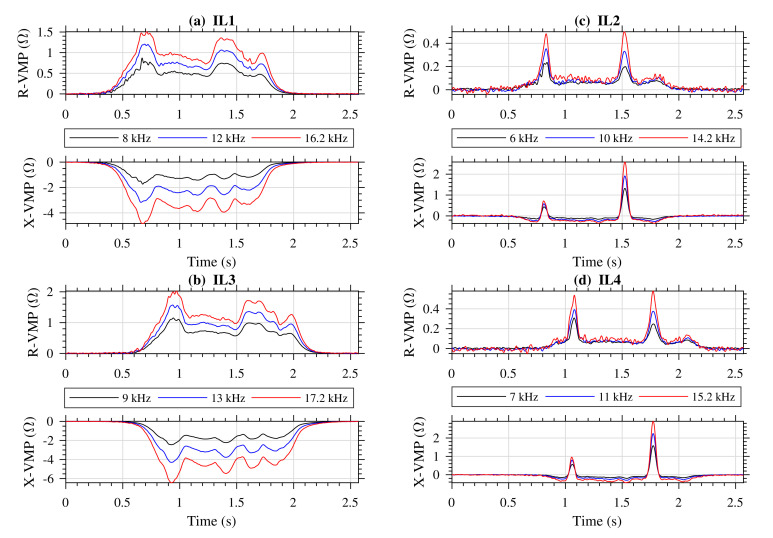
VMPs of a truck shown in Figure 7a; R-VMP and X-VMP denote the real and the imaginary impedance components; stimulation frequencies are listed in the legend; (**a**) the first standard IL1 sensor, (**b**) the second standard IL3 sensor (**c**) the first slim IL2 sensor, and (**d**) the second slim IL4 sensor.

**Figure 9:**
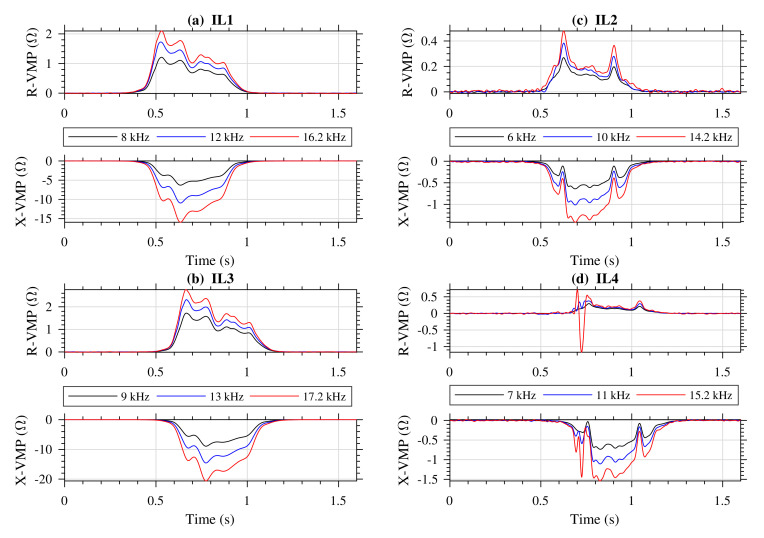
VMPs of a delivery car shown in Figure 7b, further description the same as in Figure 8.

**Figure 10:**
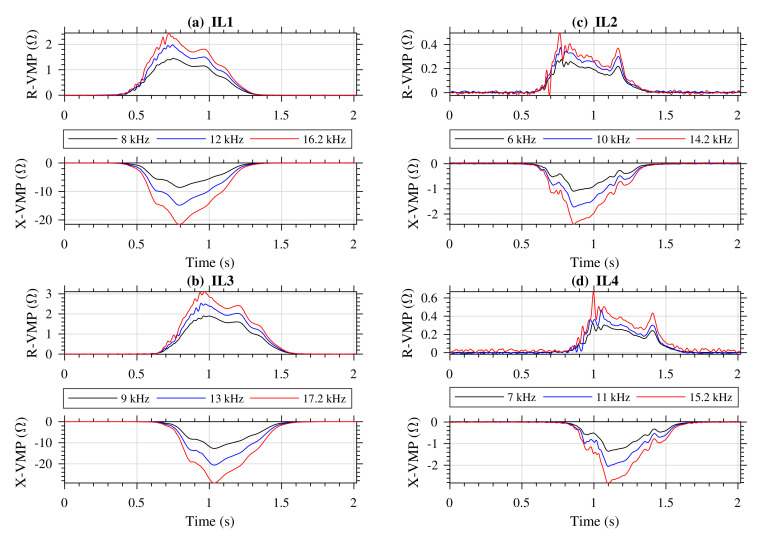
VMPs of a passenger car shown in Figure 7c, further description the same as in Figure 8.

Vehicles that passed through the sensor stand were also photographed. Figure 7 shows vehicles for which VMPs are further presented and discussed. Figure 8, Figure 9 and Figure 10 present exemplary VMPs as estimated by the FTBPHTs shown in Figure 5 for vehicles depicted in Figure 7.

Figure 8 shows VMPs of a truck presented in Figure 7a. Figure 8a presents the VMPs obtained from the first standard IL1 sensor (see Figure 1a and Figure 2), Figure 8b presents the VMPs obtained from the second standard IL3 sensor, Figure 8c presents the VMPs obtained from the first slim IL2 sensor, and Figure 8d presents the VMPs obtained from the second slim IL4 sensor, for frequencies listed in Table 4.

In the case of a truck, we conclude that (1) all R-VMPs components are positive, (2) all X-VMPs from the standard IL1 and IL3 sensors are negative, and (3) X-VMP components of slim IL2 and IL4 sensors have a characteristic spikes that comes from ferromagnetic elements in wheels, that is steel belts. Those spikes allow the measurement of vehicle axles arrangement in vehicle body and also the distance between axles.

In addition, the absolute maximum values of VMPs from the slim IL2, and IL4 are much smaller than from the standard IL1 and IL3. Due to the fact that the IL sensors are arranged one after the other on the lane, VMPs are shifted in time. Distances between IL sensors are known, therefore measurement of vehicle speed [5], and vehicle length is possible.

The VMPs of a delivery vehicle shown in Figure 7b are presented in Figure 9. It can be seen that high truck-specific spikes do not occur. Instead, there are visible subtle local maxima in VMPs from IL2 and IL4 sensors, see Figure 9c,d. Delivery vehicles generally have lower suspension than truck vehicles. Low vehicle suspension causes intense eddy currents in flat metal elements, and weakens the effect from the steel belt of a tire. R-VMP and X-VMP obtained at frequency $f_{3}$ = 15.2 kHz in channel 4 to which the IL4 sensor is connected are disturbed during interval from 0.7 to 0.8 s (see Figure 9d). A subtle local spike that comes from the vehicle axle is deformed and cannot be used in axle detection process.

The VMPs of a truck, see Figure 8, are not disturbed. The results in all channels coincide well.

The case in Figure 9d illustrates the main advantage of the proposed simultaneous multifrequency estimation. It is observed that the measurement for the excitation frequency $f_{3}$ is heavily disturbed; however, the measurement for the excitation frequency $f_{1}$ is disturbance-free. Thus, by introducing some redundancy we increased measurement reliability.

Analyzing Figure 10, that is, VMPs obtained for a passenger car presented in Figure 7c, we conclude that (1) VMPs from the standard IL1 and IL3 are different from those of the delivery vehicle, (2) VMPs from the slim IL2 and IL4 have even less visible spikes coming from the vehicle axles, (3) VMPs from the slim IL2 at $f_{3}$ = 14.2 kHz are disturbed in 0.7–0.8 s time interval, and (4) VMPs from the slim IL4 at $f_{2}$ = 11 kHz and $f_{3}$ = 15.2 kHz are also disturbed during the 0.95–1.05 s time interval. Axle detection in low-suspension passenger vehicles is the most difficult case. If there is at least one undisturbed X-VMP and one undisturbed R-VMP from the slim IL sensor, then axle detection is feasible. By using a 3-frequencies system for impedance measurement, there is increased probability of obtaining undisturbed one R-VMP and X-VMP.

Further data processing and data fusion of VMPs are expected to improve the axle counting and also the measurement of other vehicle parameters, however it is outside the scope of this work.

## 5. Conclusions

In the paper, the proposed VMP measurement system was thoroughly presented. Full system realization and operation were described. The properties of the standard and slim IL sensors and the way of their pavement installation were examined. It was practically verified that the simultaneous operation with multifrequency excitations improves the overall reliability of VMP measurement. Simultaneous measurement of VMPs at different frequencies, and processing only disturbance-free VMPs, reduces measurement system uncertainty as compared to the system working at only one frequency.

Another open problem is the possibility of retrieving the signal of interest from several possibly disturbed channels working on different frequencies.

The advantages of the described VMPs acquisition method over another methods [5,7,14,15,16] are as follows.

(1) The described method does not lose information on the real part of the IL sensor impedance and provides the R-VMP. The R-VMP and the X-VMP signals may be used in the future to develop algorithms for visualizing the vehicle undercarriage structure. It should be noted that the most commonly exploited LC-generator-based method is unable to provide the R-VMP, only the X-VMP associated with the inductance of IL sensor is acquired. On the other hand, the AC-bridge-based system provides only one VMP where the real and imaginary components of the IL sensor impedance are irreversible mixed together.
(2) The ABB method does not require time-consuming balancing and significantly simplifies the design of the analogue part which makes the system more reliable. Other methods usually exploit AC-bridge or other circuit, which requires time-consuming balancing and is generally a cumbersome problem.
(3) The described VMPs acquisition method is multifrequency and short-time. Several VMPs at selected frequencies are measured simultaneously during the short time of vehicle passage through the measurement stand. Simultaneous multifrequency measurement diminishes negative effects of a vehicle engine EMI disturbance, which, in turn, reduces the number of incorrectly measured vehicles.
(4) The described system is also a multisensor method, therefore the VMPs signal fusion is possible to be applied for more accurate or sophisticated vehicle parameters measurement.

## Figures and Tables

**Table 1:** Loops dimensions.

Dimension	A	B	C	D	E	F
**Value** (cm)	100	200	10	320	20	150

**Table 2:** Nominal IL impedance measured at 10 kHz.

Impedance Z=R+jX	Slim Loop (Ω)	Standard Loop (Ω)
loop only	0.8 + j15	0.6 + j7
loop with transformer	61 + j748	51 + j373

**Table 3:** Configuration of the digital section.

Symbol	Details
PXI	NI-PXIe-1082 Chassis, NI-PXIe-8133 Embedded
	Controller with 8 Intel Core i7 CPU Q820 1.73GHz
	and 2GB RAM.
FPGA	NI PXI-7853R (R series) - NI-RIO FPGA Device
	with 8 ADC and DAC, 750 kS/s, 16 bit, ±10 V.
Op-Amps	AD845
$R_{r}$	560 Ω, ±1%
$R_{T}$	1 Ω, ±1%
Tr	Ratio 1:7

**Table 4:** The list of excitation frequencies applied in the system.

Frequency Value in kHz in a Given Channel	$f_{1}$	$f_{2}$	$f_{3}$
#1: for the first standard IL1 sensor	8	12	16.2
#3: for the second standard IL3 sensor	9	13	17.2
#2: for the first slim IL2 sensor	6	10	14.2
#4: for the second slim IL4 sensor	7	11	15.2

where: $f_{1}$, $f_{2}$, $f_{3}$—denote excitation frequencies

## Abbreviations

The following abbreviations are used in this manuscript.

IL	Inductive Loop
ABB	Auto Balancing Bridge
IMU	Impedance Measurement Unit
VMP	Vehicle Magnetic Profile
R-VMP	Resistance VMP
X-VMP	Reactance VMP
FTBPHT	Flat Top Band Pass Hilbert Transformers
